# Inferences from tip‐calibrated phylogenies: a review and a practical guide

**DOI:** 10.1111/mec.13586

**Published:** 2016-04-20

**Authors:** Adrien Rieux, François Balloux

**Affiliations:** ^1^ Department of Genetics, Evolution and EnvironmentUCL Genetics InstituteUniversity College LondonGower StreetLondonWC1E 6BTUK; ^2^ Present address: CIRADUMR PVBMT97410Saint‐PierreLa RéunionFrance

**Keywords:** Bayesian phylogenetics, calibration, divergence time and substitution rate inferences, measurably evolving populations, population dynamics, tip‐dating

## Abstract

Molecular dating of phylogenetic trees is a growing discipline using sequence data to co‐estimate the timing of evolutionary events and rates of molecular evolution. All molecular‐dating methods require converting genetic divergence between sequences into absolute time. Historically, this could only be achieved by associating externally derived dates obtained from fossil or biogeographical evidence to internal nodes of the tree. In some cases, notably for fast‐evolving genomes such as viruses and some bacteria, the time span over which samples were collected may cover a significant proportion of the time since they last shared a common ancestor. This situation allows phylogenetic trees to be calibrated by associating sampling dates directly to the sequences representing the tips (terminal nodes) of the tree. The increasing availability of genomic data from ancient DNA extends the applicability of such tip‐based calibration to a variety of taxa including humans, extinct megafauna and various microorganisms which typically have a scarce fossil record. The development of statistical models accounting for heterogeneity in different aspects of the evolutionary process while accommodating very large data sets (e.g. whole genomes) has allowed using tip‐dating methods to reach inferences on divergence times, substitution rates, past demography or the age of specific mutations on a variety of spatiotemporal scales. In this review, we summarize the current state of the art of tip dating, discuss some recent applications, highlight common pitfalls and provide a ‘how to’ guide to thoroughly perform such analyses.

## Introduction

The idea of molecular dating was first proposed in 1962 by Zuckerkandl & Pauling (1962) when they suggested that the divergence time between two species could be measured by the number of mutations accumulated between molecular sequences (in their case, protein sequences). As molecular sequence divergence can only provide a relative timescale, calibration using an external source of information is required to convert relative into absolute divergence times. Unless one assumes the substitution rate to be known (which is a strong hypothesis irrespective of the molecular sequence under scrutiny), two sources of independent age‐related information can be exploited. One approach is to assign dates to internal nodes representing the most recent common ancestors (MRCAs) between lineages using information from the fossil record or dated biogeographical event (see box 1 in Ho *et al*. 2011a), which are rarely known with great precision. An alternative strategy, which is the focus of this review, takes advantage of the information about the age of the sequenced samples themselves to calibrate the phylogeny by assigning dates to the tips (sometimes also called terminal nodes) of the tree, hence the term tip dating. Tip dating is only possible when there is sufficient spread in the age of the samples analysed and is ideally suited for data sets of serially sampled fast‐evolving taxa, or those including ancient DNA sequences (Drummond *et al*. 2003b).

**Figure 1:**
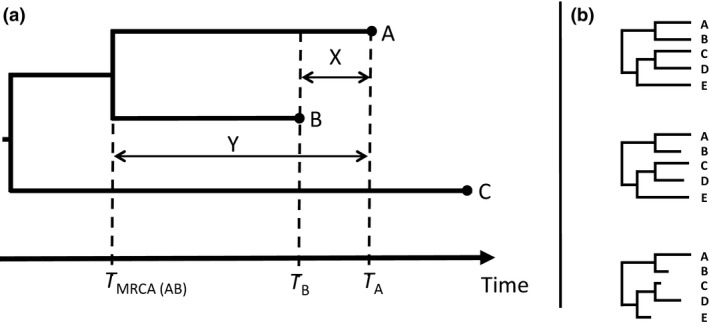
Tip‐dating principle. (a) In this simplified theoretical situation adapted from Rambaut (2000), sequences A and B were isolated at different points in time (*T*_A_ and *T*_B_, respectively) and C is an outgroup sequence. If we assume the rate of evolution to be the same in lineages A and B, then the amount of molecular evolution expected to have occurred between *T*_A_ and *T*_B_ is equal to *d*_AC_ – *d*_BC_ (*d*_AC_ and *d*_BC_ being the genetic distance between A&C and B&C, respectively). If the time X between *T*_A_ and *T*_B_ represents a significant proportion of the time Y since A and B last shared a common ancestor, then one can use tip dates to conjointly estimate the rate of evolution μ = (AC−BC)/(*T*_A_−*T*_B_) and extrapolate the age of *T*_MRCA(AB)_. (b) Top: Tree with modern samples only for which no divergence time estimate is possible without calibrations on internal nodes or a strong prior on the rate of molecular clock. Middle: Tree where tip dates may not be widely spread enough for accurate inferences. Bottom: Tree where tip date width should be sufficiently broad to allow divergence time and rate of evolution estimates with a good degree of certainty, since the sample dates cover a relatively large fraction of the total age of the tree.

The conceptual bases of tip dating were laid out in the late 1980s when sequence data from samples with associated dates of isolation started to accumulate in public databases (Rambaut 2000). In order to obtain accurate divergence time estimates for fast‐evolving genomes such as RNA viruses, the date of isolation must be accounted for. Indeed, the number of new mutations accumulated in each sequence is expected to correlate with the date of isolation. The idea of exploiting known isolation dates to conjointly estimate the rate of evolution with the time since the divergence of other internal nodes emerged by turning this reasoning around (see principle in Fig. 1). With this principle, formalized and applied to HIV data by Li *et al*. (1988), pairs of sequences must be chosen to be independent of each other by ensuring that no two pairs share any evolutionary history (branches on the tree) since their respective common ancestors. Subsequent analyses performed on hepatitis B virus data showed that the stochastic nature of the substitution process can lead to one sequence sampled earlier to exhibit more divergence from the outgroup than one sampled later and thus inflate the variance of the estimate (Bollyky & Holmes 1999). To realistically express such uncertainty, Rambaut (2000) and Drummond *et al*. (2001) introduced new methods incorporating sequence dates into a maximum‐likelihood (ML) tree reconstruction framework. Those approaches were subsequently embedded into a Bayesian statistical inference framework to jointly estimate substitution rates, divergence times and demography while accounting for the uncertainty in the genealogy by using Markov chain Monte Carlo (MCMC) integration (Drummond *et al*. 2002). Such developments ultimately led to the release of beast (Drummond & Rambaut 2007), which has been the most popular tip‐dating program developed so far (Table 1). Early implementations were assuming a constant rate of evolution throughout the tree. However, the analysis of numerous data sets showing considerable departures from clockwise evolution led to the development and incorporation of methods accounting for rate variation among lineages (Bromham & Penny 2003; Welch & Bromham 2005; Ho & Duchêne 2014).

**Table 1:** List of available and useful programs/packages to perform tip dating

Software to perform tip dating	Method	Tree topology	Models of rate variation^a^	Tip date uncertainty (as in Fig. 4)^b^	Ref/Source
beast	Bayesian	Estimated/Fixed	SC, LC, ARC, URC	a, b, c, d, e	[1]
R8s	Nonparametric	Fixed	SC, LC, DC, ARC	a	[2]
PAML (MCMCTREE)	Bayesian	Fixed	SC, ARC, URC	a, b, d, e	[3]
PHYSHER	ML	Fixed	LC, DC	a	[4]
Mr Bayes	Bayesian	Estimated/Fixed	SC, ARC, URC	a, b, d, e	[5]
TipDate	ML	Estimated	SC, DC	a	[6]
DAMBE	Distance based	Fixed	SC, DC, ARC	a	[7]
Multidivtime	Bayesian	Fixed	SC, ARC	a	[8]
LSD	Least squares	Fixed	SC	a	[9]

Software/packages to assist tip dating	Description	Ref/Source
ClonalFrameML, SplitsTree4, RDP4, LDhat	Various tools to detect recombining individuals/genomic regions	[10,11,12,13]
MapDamage2	R & Python framework to detect patterns of damage in ancient DNA reads	[14]
PartitionFinder	Python program to select best‐fit partitioning schemes and models of molecular evolution	[15]
BEAUTi	Java program assisting the preparation of infiles for running beast	[16]
TipDatingBeast	R package assisting the implementation of the date‐randomization and the leave‐one‐out cross‐validation tests in beast	[17]
SiteSampler	Java application to sample the sites of a sequence alignment to produce replicate data sets	[18]
Murray *et al*.'s R scripts	R scripts to test for temporal signal and cofounding between temporal and genetic structures	[19]
Tracer	Java program to analyse the trace (log) files generated by Bayesian MCMC programs	[20]
LogCombiner	Java program to combine log and tree files from multiple independent runs of Bayesian MCMC programs	[16]
TreeAnnotator	Java program to assist summarizing the information from a sample of treesonto a consensus tree	[16]
FigTree	Java graphical viewer of phylogenetic trees	[21]
DensiTree	Java graphical viewer for sample of (multiple) phylogenetic trees	[22]
Path‐O‐gen	Java program to plot root‐to‐tip distances as a function of sampling time	[23]
adephylo	R package to perform various phylogenetic analyses such as computing the distance of a set of tips to the root	[24]
TreeStat	Java program to calculate summary statistics (including root‐to‐tip lengths) from a set of trees	[16]
BEAGLE	High‐performance library to make use of highly parallel processors and speed up inferences	[25]
PHYLOCH	R package to read, manipulate and extract information from annotated trees	[26]

[1] (Drummond & Rambaut 2007), [2] (Sanderson 1997), [3] (Yang 2007), [4] (Fourment & Holmes 2014), [5] (Ronquist *et al*. 2012b), [6] (Rambaut 2000), [7] (Xia 2013), [8] (Thorne *et al*. 1998), [9] http://www.atgc-montpellier.fr/LSD/, [10] (Didelot & Wilson 2015), [11] (Huson & Bryant 2006), [12] (Martin *et al*. 2015), [13] (McVean *et al*. 2002), [14] (Jonsson *et al*. 2013), [15] (Lanfear *et al*. 2012), [16] Distributed in the beast package, [17] https://cran.r-project.org/web/packages/TipDatingBeast/index.html, [18] https://github.com/simon-ho/sitesampler/, [19] (Murray *et al*. 2015) [20] http://tree.bio.ed.ac.uk/software/tracer/, [21] http://tree.bio.ed.ac.uk/software/figtree/, [22] https://www.cs.auckland.ac.nz/~remco/DensiTree/, [23] http://tree.bio.ed.ac.uk/software/pathogen/, [24] (Jombart *et al*. 2010), [25] http://beast.bio.ed.ac.uk/beagle, [26] http://www.christophheibl.de/Rpackages.html.

a Models of rate variation among branches: strict clock (SC), local multirate clock (LC), discrete multirate clock (DC), autocorrelated relaxed clock (ARC) and uncorrelated relaxed clock (URC). See Ho & Duchêne (2014) for more details.

b Available distribution to model tip date uncertainty (see also Fig. 4): a: point values (no uncertainty), b: normal distribution, c: empirical description of the probability density function directly measured on the calibrated sample, d: uniform distributions with hard minimum and maximum bounds, e: uniform distribution with hard minimum and soft maximum bounds.

Concomitant with the development of increasingly sophisticated and computationally efficient phylogenetic inference algorithms during the last decade, the field of ancient DNA (aDNA) research underwent its own revolution (Millar *et al*. 2008; Der Sarkissian *et al*. 2015). Although the field's focus was initially limited to mitochondrial DNA and a few nuclear markers, numerous whole‐genome sequences from the deep past have now been retrieved. This breakthrough is tightly connected to the massive sequence throughput of modern sequencing technologies and the ability to target short and degraded DNA molecules. Additionally, the analytical power obtained through the analysis of billions of sequence reads allows quantifying contamination issues that have haunted aDNA research for decades. Whole genomes have now been sequenced from ancient anatomically modern humans (Fu *et al*. 2013a,b), archaic humans (Briggs *et al*. 2009; Krause *et al*. 2010), ancient pathogens (Bos *et al*. 2011, 2014; Rasmussen *et al*. 2015) and many animals including megafaunal species (Shapiro *et al*. 2004; Gilbert *et al*. 2008).

Following the detection and removal of potential recombining sites (see Section To date or not to date *–* when is tip dating appropriate? and Appendix S1‐A, Supporting information for more details), such time‐stamped sequence data can be integrated into phylogenetic reconstructions and used as calibration points to estimate the timing of divergence events, the rate at which substitutions accumulate and reconstruct the past demography of many species for which fossil data are not available. Tip‐based calibration represents far more than a substitute when information to calibrate internal nodes is not available, as it is expected to improve inference accuracy and robustness (Rieux *et al*. 2014; Sheng *et al*. 2014). Indeed, the uncertainty associated with the dates of time‐stamped sequences simply mirrors the uncertainty in the estimated age of the sequences (the error associated with sampling time or the C‐14 radiocarbon dating). This is a well‐characterized source of error that can be integrated into phylogenetic inference (Drummond *et al*. 2006; Ho & Phillips 2009). In contrast, calibration of internal nodes relies on the assumption of an association between an MRCA in the phylogenetic tree and information such as fossil data or biogeographical evidence. Those indirect hypothesized associations come at a cost of considerable uncertainty which is additionally much more difficult to model satisfyingly.

Besides allowing to estimating substitution rates and divergence times, thoroughly time‐calibrated phylogenetic trees represent a powerful tool for hypothesis testing. As such, it is not surprising that tip‐dating methods have been widely adopted in the fields of ancient DNA and microbial genomics (Biek *et al*. 2015). However, these methods can be complex to implement and many tip‐based inferences in the recent literature are reported with erroneously narrow confidence intervals or are even wholly incorrect. In the following, we review the current state of the art of molecular tip dating. We start with a general overview of tip‐dating methodologies and their applicability. We then review some recent results obtained through tip dating and discuss various questions that this methodology can address. Finally, we outline some future research perspectives and provide in Appendix a ‘how to’ guide to assist users performing thorough tip‐dating analyses (see major steps summarized in Fig. 5).

## To date or not to date *–* when is tip dating appropriate?

In this review, we restrict the term tip dating to analyses in which dated sequences contain sufficient temporal information for populations to be characterized as ‘measurably evolving’ (see Drummond *et al*. 2003b and description below) and can hence be used as the principal source for molecular clock calibration. Numerous studies included ancient DNA sequences from extant or extinct species into phylogenetic inference by both fixing the age of the tips (to the sample radiocarbon ages) and incorporating external information by specifying the age of one or several internal node(s) (based on the fossil record) and/or the rate of evolution (e.g. Briggs *et al*. 2009; Bunce *et al*. 2009; Rieux *et al*. 2014; Sheng *et al*. 2014; Heintzman *et al*. 2015). Among these studies, we only consider as ‘tip dating’ those that performed specific analyses to disentangle the signal coming from the different calibration sources and were able to demonstrate that tip dates had sufficient temporal spread to inform on the molecular clock (e.g. Rieux *et al*. 2014; Sheng *et al*. 2014). This definition of tip dating entails us to focus this review on studies performed at the population (i.e. intraspecies) scale. Indeed, despite recent progress in the field of ancient DNA, aDNA sequences remain generally too scarce and recent for thorough dating of ancient events (i.e. millions of years) from molecular data and tip‐dating methods alone. In this context, it is worth mentioning recent methodological developments named ‘fossil tip dating’ or ‘total evidence dating’ that allow combining morphological and molecular data to simultaneously infer the placement of a fossil in the phylogeny and calibrate trees at deeper geological times (Pyron 2011; Ronquist *et al*. 2012a). A major challenge in applying these approaches is the requisite to compile morphological character data for both extant and fossil taxa, which is complicated by the fact that for many groups the fossil record is extremely scarce and fragmentary (Arcila *et al*. 2015).

### Measurably evolving populations

Tip‐dating calibration requires working on measurably evolving populations (MEPs), a concept introduced over a decade ago by Drummond *et al*. (2003b). MEPs are populations exhibiting detectable amounts of *de novo* nucleotide changes among DNA sequences sampled at different time points. Our ability to capture measurable amounts of evolutionary change from sequence data depends on the evolutionary rate of the DNA/RNA sequence analysed (μ), its length (*L*) and the duration over which samples were collected (*t*). The pioneering research in tip dating that started around 20 years ago focused on fast‐evolving RNA viruses as at the time molecular sequences rarely exceeded 1000 bp and data sets covering sampling over 20 years were scarce (Drummond *et al*. 2003b). Progress in DNA sequencing of both modern and ancient material has led to a massive increase in both sequence length (*L*) and time span covered by the sequences (*t*). As a result, many populations from a diverse range of taxa can now be treated as MEPs (Biek *et al*. 2015).

**Figure 2:**
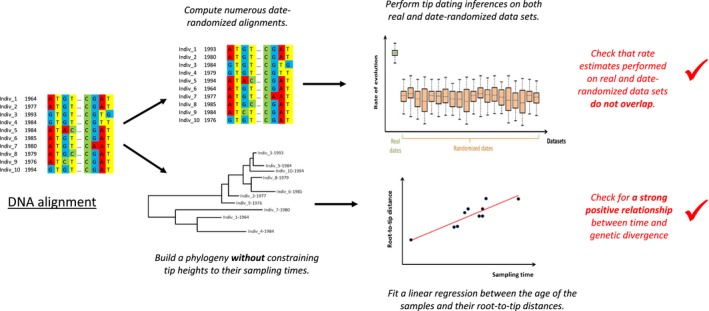
Testing for temporal signal. Flow chart for testing measurable evolutionary change in a data set prior to any tip‐dating analysis. The most robust method existing so far is the ‘date‐randomization test’ which involves generating multiple randomized data sets by permutation of sampling times, and comparing parameter estimates obtained with the initial data set *vs* the randomized ones (see Section To date or not to date *–* when is tip dating appropriate? in the text for more details on how to perform this test and interpret the results); visual evidence for a temporal signal can also be obtained by fitting a linear regression between the age of the samples and their root‐to‐tip distances, which has to be computed from a tree built without constraining tip heights to their sampling times. Different tools allowing computing date‐randomized data sets and root‐to‐tip distances are listed in Table 1.

Before performing any calibration, it is crucial to test whether there is temporal signal in the molecular data (Firth *et al*. 2010). Many studies have relied on the ‘regression method’ (Buonagurio *et al*. 1986; Shankarappa *et al*. 1999; Korber *et al*. 2000; Drummond *et al*. 2003a) which is based on the fit of a linear regression between the age of the samples and their root‐to‐tip distance (i.e. the number of substitutions separating each sample from the hypothetical ancestor at the root of the tree) (Fig. 2). Assuming statistical independence, a significant positive correlation between root‐to‐tip distances and sampling times would indicate the presence of detectable amounts of *de novo* mutations within the data set timescale. However, this is a problematic test as there is extensive pseudoreplication between samples. Indeed, the same branches in the phylogeny will contribute to multiple root‐to‐tip distances. While this problem can be partly alleviated by the use of a nonparametric test when assessing significance of the relationship, the nonindependence between distances cannot be completely controlled for (Drummond *et al*. 2003a). Additionally, the regression method can be misleading when there is substantial rate variation among lineages. The test is also likely to produce a spurious signal in the case of nonuniform distribution of the sampling times (Ho *et al*. 2007a, 2011b), which can often be the case for studies relying on aDNA for calibration where a handful of ancient sequences are typically combined with a larger number of modern samples.

A more robust method to investigate the extent of genetic and temporal signal within a data set is the ‘date‐randomization test’ (Ramsden *et al*. 2008; Duffy & Holmes 2009). This test involves generating multiple randomized data sets by permutation of sampling times, and comparing parameter estimates obtained with the initial data set vs. the randomized ones (See Fig. 2). A recent simulation‐based study (Duchêne *et al*. 2015b) recommends conducting at least 20 randomizations (this number is arbitrary and in principle it would be preferable to run a far larger number of replicates). By performing tip dating on the date‐randomized data sets, expectations of the rate and divergence time estimates can be computed in the absence of temporal signal. To determine whether there is sufficient temporal signal in a data set, one thus needs to verify that the mean rate (or TMRCA) estimated with the original sampling times is not contained within any of the 95% credible interval of those obtained from the date‐randomized data sets. A more stringent criterion is to compare the 95% credible interval of the original rate (or TMRCA) instead of its mean. Importantly, the ‘date‐randomization test’ can accommodate nonrandom sampling resulting in a nonuniform distribution of sampling times by a modification of the randomization procedure. The latter involves identifying clusters of samples with the same, or very similar, sampling times and permuting the sampling times among but not within these clusters (Duchêne *et al*. 2015b).

Finally, a distinct approach uses model selection and compares the fit of models with the sampling dates included or excluded (Rambaut 2000; Drummond *et al*. 2003a; Baele *et al*. 2012). Temporal signal is confirmed if the inclusion of the sampling dates improves the fit. In practice, to keep the two cases as similar as possible, tip dates for the ‘no dates’ model are set to the most recent sampling date in the original data set (Murray *et al*. 2015).

Two recent studies investigated the performance of several of the above tests using both simulated and empirical data sets. Murray *et al*. (2015) showed that all of the standard tests of temporal signal can be misleading for data where temporal and genetic structures are confounded (i.e. where related sequences are more likely to have been sampled at similar times). Duchêne *et al*. (2015b) demonstrated that the performance of the date‐randomization test can be affected when the sampling times are not uniformly distributed throughout the tree. On a more positive note, both studies show that the ‘clustered permutation’ date‐randomization approach can successfully correct for such confounders. Consequently, we highly recommend performing the clustered date‐randomization test using the most stringent criterion prior to any study aiming to applying tip calibrations. While the regression approach is statistically unsatisfying, it can still be used as a visual ‘sanity check’ for the reliability of rate estimates since the slope coefficient corresponds to the substitution rate under the assumption of a strict molecular clock, and the *R* ^2^ coefficient of determination indicates the degree to which sequence evolution followed a clocklike rate. For data sets failing to pass the DRT, the sampling and/or the sequencing strategy should be modified to widen the sampling time window (e.g. by including older samples, if possible) and/or increasing the number of sites in the alignment (e.g. by sequencing more nucleotides, if possible). Investigating the temporal signal of a data set is crucial because in the absence of a temporal signal in the data, the result will be driven by the prior, and is thus likely to be misleading.

### Nonrecombining sequences

Central assumptions behind all phylogenetic models developed so far are that sequences evolve neutrally, and have not undergone genetic recombination. Although deviation from neutrality can satisfyingly be accommodated (see Appendix S1‐C, Supporting information), accounting for recombination in phylogenetic inferences remains a difficult task and ignoring it leads essentially systematically to misleading inferences (Hedge & Wilson 2014). The rate of homologous recombination has been shown to vary significantly between species (Smukowski & Noor 2011), between lineages within species (Didelot *et al*. 2012; Bauer *et al*. 2013) as well as between regions within genomes (Everitt *et al*. 2014; Yahara *et al*. 2014). The influence of recombination will hence vary from data set to data set. In addition to its potential to distort tree topology, recombination can also create a false signal of apparent mutational evolution by introducing additional divergence between samples taken at different time points (Sanchez‐Buso *et al*. 2014). The easiest strategy to avoid such bias when analysing MEPs is to consider recombination‐free genomic regions such as mitochondrial DNA. An alternative approach is to perform a preliminary analysis to detect recombination events in the alignment based on conflicts in the topologic placement in the phylogeny and exclude incriminated individuals and/or genomic regions from subsequent dating inferences or partition the data around recombination breakpoints (see Appendix S1‐A, Supporting information).

## Applications of tip dating: hypotheses testing and potential sources of errors

Once nonrecombining genomic regions have been identified, tip‐dating inferences can be performed to test various hypotheses while estimating evolutionary rates and timescale‐related parameters. This usually involves considering phylogenetic methods based on molecular clocks, which make assumptions about patterns of rate variation among lineages (see Ho & Duchêne 2014; Welch & Bromham 2005 for previous reviews on this particular topic). In the following, we discuss various recent tip‐dating applications.

### Estimating the age of internal nodes in a phylogenetic tree

One of the most popular aims of dating analyses is to convert the genetic divergence measured between sequences of DNA into an absolute age for any internal node of a phylogenetic tree. This estimated node age is referred to as the time to the most recent common ancestor (TMRCA). Internal phylogenetic nodes represent such putative ancestors for all sampled individuals within a clade defined by a node and can as such sometimes be associated with key chapters in a species’ evolutionary history. In humans for instance, dating events such as the dawn of humankind millions of years ago or the expansion of anatomically modern humans from an African cradle some 100 k years ago have received tremendous interest. By using mitochondrial genomes from ancient archaic and modern humans spanning 65 000 years as calibration points for the mitochondrial clock, recent studies were able to date the divergence between Chimpanzee, *Homo neanderthalensis* and *H. sapiens* as well as the TMRCA of all modern human mtDNAs with an unprecedented precision (Fu *et al*. 2013b; Rieux *et al*. 2014). Also, by assuming that the coalescence of certain candidate haplotypes coincides with discrete human expansion or migrations, tip‐dating analyses allowed to date various other events such as the ‘out of Africa’ exit or the initial colonization of different islands such as Sahul, Japan, Remote Oceania, Madagascar, New Zealand (Rieux *et al*. 2014).

Beyond simply reflecting a divergence event between lineages and taxa, age estimates for internal nodes can also provide information about the timing of emergence of novel specific genetic variants (Holden *et al*. 2013; Spagnoletti *et al*. 2014). For example, Eldholm *et al*. (2015) used whole‐genome sequences of the nonrecombining bacteria *Mycobacterium tuberculosis* (*Mtb*) obtained from isolates sampled over 13 years to reconstruct the dynamic of antibiotic resistance‐conferring mutations during a major ongoing outbreak in Argentina. Their results indicate that a multidrug‐resistant *Mtb* strain had been circulating for 15 years before the outbreak was initially detected and about one decade before the earliest documented transmission of *Mtb* strains with such extensive resistance profile.

Dating internal nodes can also provide meaningful information about pathogen host species jumps such as those leading to novel emerging zoonotic, agronomic and wildlife diseases (Parrish *et al*. 2008; Engering *et al*. 2013). By using a panel of whole‐genome sequences of *Staphylococcus aur*eus representing the breadth of the species’ diversity with known dates of isolation, Weinert *et al*. (2012) were able to infer and map the different host switches on the phylogenetic tree while accounting for uncertainty about ancestral host associations. Their results point to multiple jumps back and forth between human and bovids with the first switch from humans to bovids dating back to around 5500 BP, which coincides with the time when cattle domestication started expanding throughout the Old World, thus suggesting a central role for anthropogenic change in the emergence of new endemic diseases.

Estimation of evolutionary timescales from tip‐calibrated phylogenies has become routine in biology, forming the basis of a wide range of evolutionary and ecological studies as illustrated above. However, it is important to remember that various sources of error can affect these estimates, including incorrect calibration dates (Ho *et al*. 2008; Molak *et al*. 2013), misspecification of the demographic, substitution and molecular clock models (Navascues & Emerson 2009; Ho *et al*. 2011b; Duchêne *et al*. 2015c) or the presence of tree imbalance (Duchêne *et al*. 2015a). Whereas the effect of model misspecification is a common issue in biology, the influence of tree imbalance is worth considering as in addition to being affected by biological factors such as past demography or natural selection, it is also directly related to sampling effort and strategy. Tree imbalance refers to the relative number of tips descending from internal nodes in a phylogenetic tree. In a completely balanced tree, each of the two lineages descending from any internal node leads to the same number of tips. A completely imbalanced tree has a pectinate or comb‐like arrangement of branches. Imbalanced ultrametric trees are characterized by an excess of long branches because some of the lineages will have few descendants in the sample. In their study based on both simulated and empirical data sets, Duchêne *et al*. (2015a) found that tree imbalance had a detrimental impact on dating precision and produced a systematic bias with overall timescales being underestimated. A pronounced effect was observed in analyses with shallow calibrations. The greatest decrease in accuracy usually occurred in the age estimates for medium and deep nodes of the tree. Those results indicate that in case of tree imbalance, molecular clock analyses can be improved by increasing taxon sampling, with the specific aims of including deeper calibrations, breaking up long branches and reducing tree imbalance.

### Estimating rates of evolution and their variation

Conjointly with the estimation of the TMRCA of two or more sampled genomes, dating a phylogenetic tree also allows inferring the rate at which mutations accumulate on their connecting lineage (Fig. 1). Substitution rates can be heterogeneous both between genomic regions (Bromham & Penny 2003) and along evolutionary lineages (Lanfear *et al*. 2010). Various partitioning algorithms allow accounting for heterogeneity within genomes (e.g. Lanfear *et al*. 2012) and dedicated molecular clock models have been developed to deal with variable substitution rates along lineages (see Ho & Duchêne 2014; Welch & Bromham 2005 for more details on this topic).

Such developments allowed, for example revising the rate at which the human mitochondrial DNA accumulates mutations (Fu *et al*. 2013b; Rieux *et al*. 2014). By pre‐estimating the optimal partitioning scheme and the best‐fit nucleotide substitution model for each partition of the mtDNA molecule (see Appendix S1‐C, Supporting information), and taking advantage of the wealth of sequenced ancient human mitochondrial genomes from the last 65 000 years, it was possible to refine the mitochondrial substitution rates for different genomic regions and codon positions (Rieux *et al*. 2014). In addition, the use of a relaxed molecular clock allowed investigating rate variation along evolutionary lineages. There is an extensive debate in the literature on the existence and significance of time‐dependent rates of molecular evolution (Ho *et al*. 2011a). In particular, one common observation is for substitution rates to accelerate in recent generations in several species including humans (Ho *et al*. 2007b, 2011a; Henn *et al*. 2009; Duchêne *et al*. 2014). This phenomenon is generally ascribed to the time needed for natural selection to weed out deleterious mutations from the population. Consistent with this pattern, the recent rates obtained for human mitochondrial DNA recovered a subtle but significant negative correlation between the age of the ancient genome used for calibration and the substitution rate estimated (Rieux *et al*. 2014). Stronger evidence for purifying selection was found in the form of a stark difference in the substitution rates of first and second codons (PC1 + 2) versus third codon (PC3). Although PC3 mutations accumulate linearly with time in humans, a clear acceleration in the rate starting at around 30 000 years was recorded for the mostly nonsynonymous mutations at PC1 + 2 (Rieux *et al*. 2014).

In addition to allowing to explore patterns of rate variation in time, tip dating combined with the relaxed molecular clocks also permits investigating variation in substitution rates across lineages. One of the clearest evidence for dramatic heterogeneity in substitution rates has been reported for historical variation in substitution rate in *Yersinia pestis*, the aetiologic agent of plague (Cui *et al*. 2013) with nearly 40‐fold difference between the slowest and the fastest evolving branches.

Like for any model‐based inference procedure, inference quality of tip‐based Bayesian coalescent analyses will depend on how well the model captures the underlying processes that generated the data. In this context, it has been shown using simulations that molecular rate estimates obtained from ancient DNA‐based calibrations can be upwardly biased for populations evolving under complex demographic scenarios and/or populations with large effective sizes, as both situations tend to produce genealogies where ancient and modern DNA samples segregate in different lineages (Navascues & Emerson 2009; Emerson *et al*. 2015).

### Reconstructing past demography

Conjointly with rates of evolution and divergence times, effective population sizes (EPSs) through time can be estimated from a time‐calibrated tree using Bayesian skyline plots (BSP) and related methods implemented in coalescent‐based algorithms such as beast (see Ho & Shapiro 2011 for a recent review). The EPS corresponds to the number of idealized individuals that contribute offspring to the descendent generation and is almost always smaller than the census population size. One interesting feature of such reconstruction methods is that they are well suited to detect population bottlenecks. Such estimates of EPS variation have notably allowed investigating the relative impacts of climatic and anthropogenic factors on the widespread extinctions of large mammals such as the steppe bison (Shapiro *et al*. 2004) or the cave bear (Stiller *et al*. 2010) at the end of the Pleistocene epoch.

Skyline‐plot‐based demographic inferences also intensively contributed to the field of molecular epidemiology since EPS plots can help detecting when epidemics took off. In a recent study published thirty years after the discovery of HIV‐1, Faria *et al*. (2014) made use of more than 800 concatenated RNA sequences obtained from individuals infected by the virus between 1959 and 2004 in Africa and the USA to shed light on the early transmission, dissemination and establishment of the virus in human populations. Their findings indicate that Kinshasa in the 1920s was the epicentre of early transmission and the source of pre‐1960 pandemic viruses elsewhere and that the demographic history of group M and nonpandemic group O was similar until ~1960, when the M group underwent an epidemiological transition and outpaced regional population growth. Their results emphasize the likely role of iatrogenic interventions and changes in sexual behaviour in the emergence of HIV‐1 group M. In addition to shedding light on the origins of an epidemic, the combination of tip dating and demographic reconstruction also allows testing whether policy changes for managing an epidemic have been effective. This has notably been the case with the United Kingdom HIV‐1 epidemic and Egyptian hepatitis C virus (HCV) epidemic (Stadler *et al*. 2013), 2009 influenza A (H1N1) pandemic (Fraser *et al*. 2009) or 2014–2015 Ebola epidemics (Stadler *et al*. 2014).

The use of BSPs and related methods for the estimation of past EPS has become increasingly common in the literature, covering a wide spectrum of taxa from viruses to large mammals. Despite their appeal, EPSs are not always straightforward to interpret and to equate to actual census sizes (Frost & Volz 2010). It is also important to remember that BSP models are assuming a single panmictic population and violation of this assumption can lead to misleading inferences (Stack *et al*. 2010; de Silva *et al*. 2012; Heller *et al*. 2013). Damage in ancient DNA has also been shown to potentially distort inferences of demographic histories (Axelsson *et al*. 2008; Rambaut *et al*. 2009). Particular attention should thus be given to those factors in future studies making use of BSP models to study past demography (more details in Appendix S1‐B, Supporting information).

### Estimating the age of a sample

Another application of tip‐dating analyses is the estimation of the age of a sample for which this information is missing using phylogenetic molecular clock‐based methods. There are a number of circumstances under which the leaf ages of sequences may be unknown or at best, highly uncertain. First, ancient DNA sequences may be amplified from specimens older than the 50–55 000 years BP radiocarbon limits. For example, nearly 100 of the bison sequences reported in Shapiro *et al*. (2004) were too old to be assigned finite radiocarbon ages. Second, for rapidly evolving taxa, the date of sampling may be unknown due to the loss or absence of accurate archival information. Even if the sampling date is known to the nearest year, it may be critical to estimate the isolation date more accurately. Finally, it may also be important to independently assess the authenticity of posited sampling dates due to their extreme age or because they are contentious.

Shapiro *et al*. (2011) developed and implemented a leaf‐dating method that estimates the age or date of isolation of individual sequences within the Bayesian MCMC framework provided by the software package beast (Drummond & Rambaut 2007). In this method, the sequence's leaf age is treated as a random variable and an additional parameter for the age of the terminal node is introduced and treated identically to the internal node age parameters in terms of proposals made by the MCMC kernel. Methodologically, the leaf‐dating method is similar to relaxing the constraints of the molecular clock on specific lineages within a phylogenetic tree. The method has been validated using both simulated and empirical data sets (Shapiro *et al*. 2011) and applied to different data sets to estimate the unknown age of some specimens before integrating them into classical tip‐dating analyses (Gray *et al*. 2013; Stadler *et al*. 2013; Alter *et al*. 2015). For example, Faria *et al*. (2014) included a historical HIV strain from 1959 as an internal control and estimated its age, recovering an estimate centred on 1958 (95% BCI: 1946–1970). Although the estimated ages recovered are often associated with wide credible intervals, the leaf‐dating method provides a means to include in molecular clock analyses sequenced samples for which little or no temporal information is available. Incorporating additional sequence data can improve the resolution of the phylogenetic, demographic and geographic history of the sampled sequences and can significantly extend the temporal range of the analysis.

### Reconstructing transmission chains

**Figure 3:**
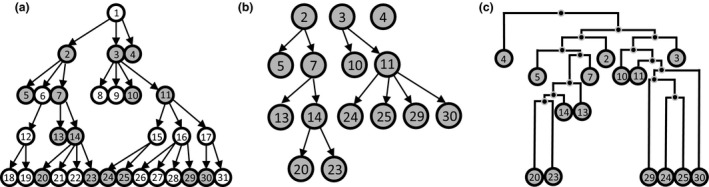
Transmission graph vs. Phylogenetic tree. This figure adapted from Jombart *et al*. (2011) illustrates the difference between a transmission chain and a phylogenetic reconstruction. Panel a represents the transmission chain of a pathogen as arrows connecting hosts represented as circles, with grey circles representing sampled hosts. In panel (a, b) transmission graph (or network) is correctly reconstructed from the sampled hosts. In panel (c), a time‐structured phylogeny is reconstructed using the same samples with black dots representing hypothetical ancestral isolates.

Reconstructing transmission chains (i.e. who infected whom) using phylogenetic reconstruction methods has received considerable interest over recent time (Croucher & Didelot 2015). Although it should be stressed that while a phylogenetic tree and a transmission chain are both mathematical graphs representing the sequences as nodes connected by edges, they have radically different properties and are not interchangeable (Fig. 3) (Jombart *et al*. 2011; Didelot *et al*. 2014; Hartfield *et al*. 2014). Phylogenetic trees are binary graphs where the samples occupy the tips (terminal nodes) with each pair of samples connected by one node representing their putative most recent common ancestor (MRCA). Conversely, transmission chains are networks where each sample is represented by one node (internal or terminal) that can be connected to an arbitrary number of other nodes by edges representing between‐host transmission events of the pathogen.

Phylogenetic reconstruction can in some circumstances inform on the transmission chain. However, a naïve phylogenetic analysis can lead to highly inaccurate inference of transmission under a series of realistic scenarios. If sampling is shallow so that only a small proportion of infected individuals are sampled, isolates connected in a phylogenetic framework are indirectly linked via a chain of unsampled hosts, and the branches in the tree will not represent direct transmission events. Conversely, in the case of densely sampled outbreaks and epidemics, another problem arises as some samples can be both ancestors and descendants of other samples. This pattern is not well captured by a classical phylogenetic tree which considers all samples as descendants (tips) connected by inferred ancestors (nodes) (Fig. 3c). Additional problems arise for pathogens that remain infectious over a period of time (i.e. chronic infections), when a pathogen lineage continues to evolve within a host after the latter has infected other individuals. Finally, it has been shown that in the presence of within‐host pathogen genetic diversity, a hallmark of most viruses and an increasingly widely recognized pattern in bacteria alike, phylogenetic reconstruction struggles to inform on the transmission chain (Worby *et al*. 2014).

While these represent major challenges, the field has seen some recent exciting developments aiming at adapting tip‐based phylogenetic reconstruction to the reconstruction of transmission chains. Didelot *et al*. (2014) introduced a Bayesian inference scheme which allows superimposing the transmission network upon a phylogenetic tree applicable to well‐sampled outbreaks while accounting for within‐host evolution. Also of note, is the recently developed Bayesian MCMC algorithm by Gavryushkina *et al*. (2014) to infer sampled ancestor trees, that is trees in which sampled individuals can be direct ancestors of other sampled individuals. When analysing an HIV data set from the United Kingdom, they were able to detect sampled ancestors and estimate the probability that an individual will be removed from the process upon sampling (i.e. diagnosis). They could also demonstrate that even if sampled ancestors are not of specific interest in the analysis, failing to account for them leads to significant bias in the branching model and clock rate estimates.

## Concluding remarks and future perspectives

Introduced over a decade ago, the concept of measurably evolving populations (MEPs), along with the analytical methodology it has spawned, has revolutionized our ability to study population dynamic and evolutionary processes using genetic data. Although only fast‐evolving taxa such as RNA viruses were initially classifiable as MEPs, the recent rise in our ability to sequence DNA at high throughput both from modern and ancient material has opened up the field to a variety of additional organisms (Biek *et al*. 2015). Crucially, the sampling dates need to have sufficient temporal spread to capture measurable amounts of evolutionary change and perform tip dating thoroughly. In this context, we hope this review will encourage users of tip‐dating methodologies (Fig. 5) to systematically investigate and report the temporal signal existing in their data set (see Section To date or not to date *–* when is tip dating appropriate? and Appendix S1‐D, Supporting information) to avoid rate and divergence time estimates to be mostly driven by prior information. We also hope to see more studies investigating the performance of the currently available tests to identify MEPs, such as the ones recently performed by Duchêne *et al*. (2015b) and Murray *et al*. (2015). Compelling directions for future research may target data sets with large number of modern sequences and a small set of ancient sequences, for which the date‐randomization test might not be appropriate.

**Figure 4:**
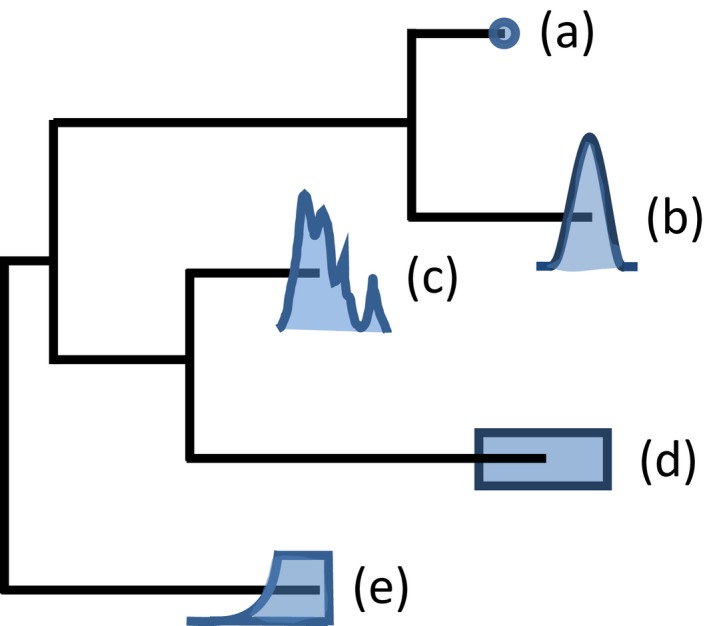
Different statistical distributions to model uncertainty in tip calibrations inferences. Different distributions can be used to model the error associated with sampling dates. Choosing the best‐suited one depends on the type of sample and the information associated with the dating method (Ho & Phillips 2009). Point values (a) can be used if the age of a sample is exactly known (e.g. sampling date). Modelling radiocarbon dating errors with a normal distribution (b) is common practice in ancient DNA studies even though recent improvement allow to use empirical description of the probability density function directly measured on the calibrated sample (c) (see Molak *et al*. 2015 for more details on this topic). Uniform distributions with hard minimum and maximum bounds (d) are suited to samples obtained from a well‐defined stratum [e.g. ancient DNA retrieved from ice cores (Willerslev *et al*. 2007) or from samples associated with archaeological horizons (Edwards *et al*. 2007)] or to model uncertainty in sampling time accuracy (e.g. if the sampling month is known for some samples but not for others). Finally, uniform distribution with hard minimum and soft maximum bounds (e) can be suited to ancient DNA samples beyond the 45–50 ka resolution limit of radiocarbon dating (thus yielding a minimum age) for which additional information (e.g. from fossil data) exists and justifies the use of a soft maximum bound. This figure is adapted from Ho & Duchêne (2014).

**Figure 5:**
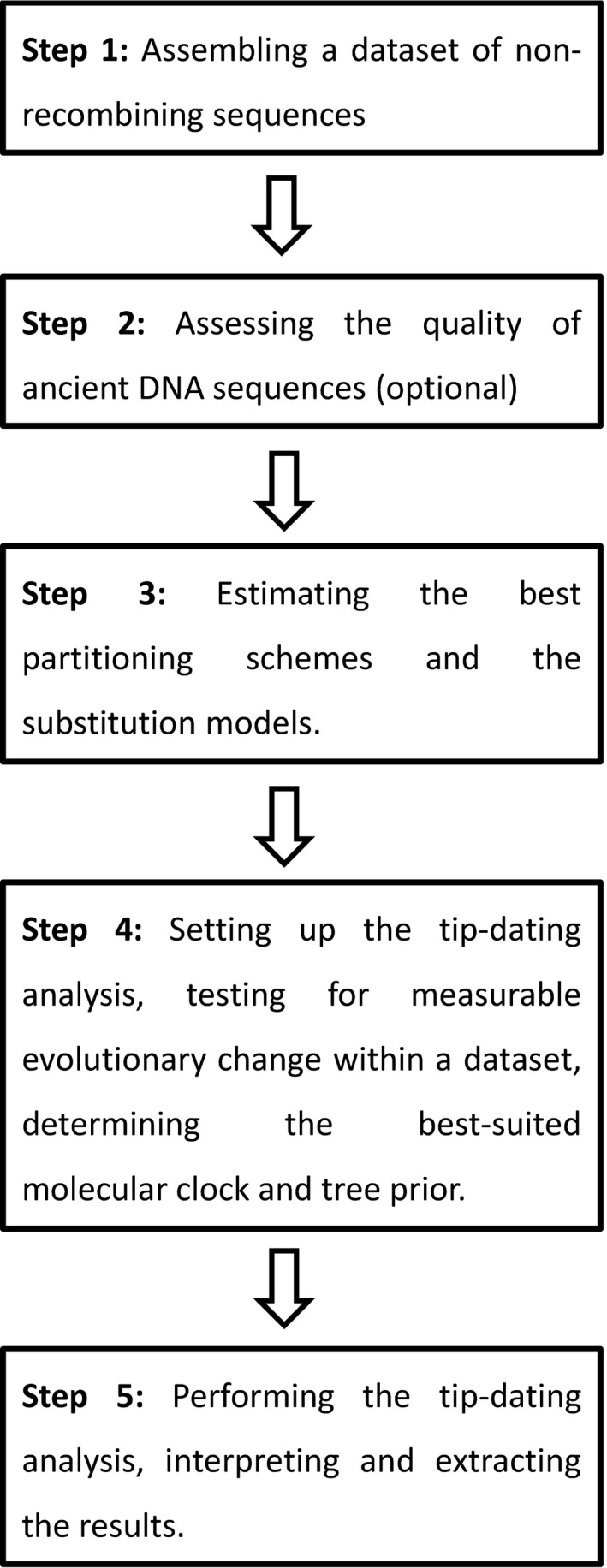
Major steps to conduct accurate tip dating. This figure summarizes the five main steps that ought to be conducted when performing tip‐dating analyses. For each of those steps, additional advices such as the important choices that must be made or the software to be used are given in the form of a practical guide available in Appendix S1 (Supporting information).

It is important to acknowledge that substantial advancements have been made in the field of molecular dating using heterochronous samples. Since the first attempts of incorporating noncontemporaneous sequences into maximum‐likelihood (Rambaut 2000) and Bayesian (Drummond *et al*. 2002) frameworks made 15 years ago, many refinements have allowed for improved inference in a variety of biological systems. Those comprise relaxed molecular clocks, more realistic demographic models including the Bayesian skyline plot, the possibility to explicitly model the error associated with sampling times and the accumulation of post‐mortem damage of DNA with time. Current molecular‐dating models still fail to fully allow for joint reconstruction of the phylogeny and recombination patterns even though progress is being made towards that direction (McGill *et al*. 2013; O'Fallon 2013). However, such ancestral recombination graph (ARG) models will likely always be limited to situations where the rate of genetic recombination remains low as the phylogenetic paradigm is in essence not applicable to taxa undergoing extensive recombination. Additional compelling directions for future refinements may focus on spatially explicit modelling of population structure as the latter has been shown to be a factor distorting evolutionary rate and divergence time estimates. Finally, computational developments allowing for an improved use of highly parallel processors are also required to reduce calculation time that can be enormous when dealing with parameter‐rich models and huge genomic data sets.

A.R. and F.B. contributed equally to the writing of this review.

## Supporting information


**Appendix S1.** A step‐by‐step practical guide to conduct accurate tip‐dating.
**Appendix S2.** Manual modifications to perform to the xml‐input files.
