# Supplementary material for: Inferences from tip‐calibrated phylogenies: a review and a practical guide
Source: Mol Ecol. 2016 Apr 20;25(9):1911–24. doi: 10.1111/mec.13586 (PMC4949988; doi:10.1111/mec.13586)
Supplement: Supplementary file 1 — Appendix S1. A step‐by‐step practical guide to conduct accurate tip‐dating. Appendix S2. Manual modifications to perform to the xml‐input files. [file MEC-25-1911-s001.docx]

**Supplementary material**

**1- A step-by-step practical guide to conduct accurate tip-dating**

Conducting a thorough tip-dating analysis is a challenging endeavor that generally requires substantial amount of time and taking advantage of several programs to be used in a back and forth manner. In this document we outline some of the important choices that must be made when performing tip-dating analyzes. We have chosen to describe the main inferences taking the software BEAST as example because of its flexibility and its popularity but similar investigations can be performed with alternative tools (as listed in Table 1 of the main document). The whole process of tip-dating can be divided into five major steps detailed below:

**A) Assembling a dataset of non-recombining sequences**

As phylogenies determine the genealogy of sequences based on the number of differences between them, a set of aligned sequences is required to feed any tree-based analysis. There are many programs to align DNA sequences. We will not discuss them here but care must be taken to ensure there are n o misalignments and few gaps. In BEAST, gap characters ('-', '?' or 'N') are treated as missing, meaning they do not contribute to the likelihood for that branch and site (i.e. there is equal marginal probability for the 4 nucleotides). Simulations and empirical analyses suggest that missing data is generally not a major issue for sufficiently long sequences (>100 characters) even for very high fractions of missing data (up to 95%) (Drummond & Bouckaert 2015; Wiens & Moen 2008). If possible, the closest outgroup to the clade of interest should be included to facilitate rooting the tree but this is not mandatory since the program can search for the most likely position of the root in the tree without an outgroup. In BEAST, it is possible to perform most of the actual dating analyses using the variant nucleotide sites (SNP) only (as shown in part 2a of this document), but the whole sequences (variants + invariants sites) are required for preliminary investigations with other programs. If recombination has occurred between sequences, the tree topology will be distorted and software such as LDHat (McVean *et al.* 2002), RDP (Martin *et al.* 2015) , SplitsTree4 (Huson & Bryant 2006) or ClonalFrameML (Didelot & Wilson 2015) will be needed to detect recombining individuals and/or genomic regions. These can either be removed, or the alignment split at the recombination point, with each part analyzed separately.

**B) Assessing the quality of ancient DNA sequences (optional)**

This step is required if ancient DNA samples are included in the dataset. It is well documented that substantial chemical modifications of nucleotide bases is introduced *post mortem* as a result of DNA damage (Paabo 1989; Sawyer *et al.* 2012). Such damage leads to specific signatures of nucleotide mis-incorporations and DNA fragmentation in sequencing reads, which can be used to argue that the reads are genuinely from ancient remains rather than modern contaminants (Green *et al.* 2008). However, damaged DNA may also lead to misleading inferences of rates of evolution (Rambaut *et al.* 2009) and demographic histories (Axelsson *et al.* 2008). When working with ancient DNA, it is thus critical to assess the quality of the sequences prior to any tip-dating analysis (Der Sarkissian *et al.* 2015). Patterns of damage in ancient DNA can be detected either from sequencing reads using *ad hoc* programs such as mapDamage2 (Jonsson *et al.* 2013) or from concatenated sequences by quantifying an excess of A/T *vs* G/C singleton SNPs as described in Rieux *et al.* (2014). If only a small proportion of ancient samples are affected, we suggest excluding them from subsequent analyses. If damage is pervasive or affects samples critical for the analysis, we recommend using a model in which sequences are the result of a joint process of molecular evolution and post mortem DNA damage, as implemented in BEAST by Rambaut and colleagues (2009). Another strategy could be to simply remove all sites involving transitions from ancient sequences prior to analysis as done in Axelsson *et al.* (2008) but this procedure has been shown to result in loss of power to detect patterns of population change (Rambaut *et al.* 2009).

At this stage of the analysis, the date of sampling of each molecular sequence in the dataset should be known or estimated (Shapiro *et al.* 2011) as described in section 2.4 of the main document. In the FASTA file, we recommend including the age of each sample (along with any other insightful information such as the sampling location) into the corresponding sequence name to facilitate setting up the tip-dating analysis in step D. Ages can be in any unit of time (e.g. years, months or days) as long as the same unit is used for all samples.

**C) Estimating the best partitioning schemes and the substitution models.**

Mutation is often heterogeneous between different genomic regions. Partitioning involves splitting sites in an alignment into sets that have evolved under similar processes. Choosing an appropriate partitioning scheme is an important step in phylogenetic analyses because it can affect the accuracy of tip-based inferences as well at their associated errors. The program Partitionfinder (Lanfear *et al.* 2012) allows for the combined selection of best-fit partitioning schemes and nucleotide substitution models, which describe how mutations arise between related samples over time. To speed-up the analysis, it is possible to provide pre-defined data blocks (e.g. different genes or nucleotide types). For instance, in a study focusing on whole human mtDNAs Rieux *et al.* (2014) defined the following seven groups of nucleotides: 1) HVS1, 2) HVS2, 3) Protein coding (PC) positions 1, 4) PC2, 5) PC3, 6) tRNAs and 7) rRNAs. Partitionfinder thus analyzed every scheme that includes those seven groups in any possible combination. Best partitioning was obtained for K = 4 schemes (PC1+2, PC3, HVS1+2 and r+tRNA), with each partition being assigned its best model of evolution. When the dataset includes ancient sequences, particular attention should be paid to verifying that the combined selection of schemes and substitution models is not driven by patterns specific to the ancient DNA samples. One strategy to account for this relies on running Partitionfinder both with and without the ancient sequences.

**D) Setting up the tip-dating analysis, testing for measurable evolutionary change within a dataset, constant molecular clock and determining the best tree prior.**

The BEAUti program is a tool assisting the user in creating the XML-formatted input file required to perform a tip-dating analysis with BEAST (see for example the “Using sequences sampled at different points in time to estimate rates in dengue virus” tutorial available from <http://beast.bio.ed.ac.uk/tutorials>). Setting-up a basic tip-dating analysis consist in calibrating the molecular clock with tip dates only. This means that other sources of information that can sometimes be used for the same purpose, such as the rate of evolution or the age of internal nodes must not be incorporated at this stage. In practice, this is done by fixing the height of the terminal nodes in the tree (i.e. tips) to their respective ages and using non-informative priors (i.e. flat distributions) for the rates of evolution and the ages of the internal nodes (which includes the root of the tree). Fixing the height of the terminal nodes to the age of the sample can be done in BEAUTi (by parsing the sampling dates from each sample name in the tips section). Dates can be specified relative to a point in the past (as would be the case for calendar years) or backwards in time from the present (as in the case of radiocarbon ages). Uncertainty about sampling time must be incorporated if available. Different distributions (uniform, normal, gamma…) can be used to model the error (see Figure 4 in the main document and Ho & Phillips 2009 for more details).

Once set up, the first step of the tip dating analysis requires choosing a molecular clock model and the best suited tree prior model, which describe how coalescence events are distributed over time. For the molecular clock, a common strategy is to test for deviations from a strict clock by considering the standard deviation of the uncorrelated lognormal relaxed clock (the parameter ucld.stdev). If this parameter takes a small value (close to 0), there is little variation in rates among branches, and the sequence data can be considered to have evolved in a clock like manner. If this parameter takes values greater than 1 then the standard deviation in branch rates is greater than the mean rate, and the data exhibits substantial rate heterogeneity among lineages. As highlighted in a recent study using posterior predictive simulations, evaluating the absolute performance of molecular clock models is a critical step to ensure a good fit between the data and the clock-model selected (Duchêne *et al.* 2015). For the tree prior, BEAST integrates two main types of models reflecting two levels at which the biological processes responsible for generating the pattern of lineage divergences can be described. The first category contains coalescent models describing population-level processes (e.g. Bayesian skyline-plots methods, constant size or exponential growth). Such models are suited to analyze datasets mostly composed of individuals from the same population or species. Separate coalescent models can be applied to different clades of a phylogenetic tree in a single BEAST analysis as done previously (Ho *et al.* 2008; Rieux *et al.* 2014) and described in part 2b of this document. It can be wise to start by considering one of the implemented skyline-plot methods to get an idea whether the population size is more likely to be constant or changing over time. The second category of tree prior models contains stochastic branching models describing lineage diversification at the species level (e.g. Yule, birth-death, the calibrated Yule (Heled & Drummond 2012), birth-death with incomplete species sampling (Stadler 2009), serially-sampled birth-death processes (Stadler 2010) and the fossilized birth-death models (Heath *et al.* 2014)). Those models should be used when most samples belong to different species. More extensive investigations should be performed by launching different analyses under contrasted assumptions and comparing the performance of different models (e.g., strict *vs* relaxed molecular clock or constant size *vs* exponential growth) using Log-likelihood statistics along with the obtained parameter estimates. Model choice can be based on Bayes factors calculated from the marginal likelihoods of alternative models, as recommended by Baele *et al.* (2012).

Once both the molecular clock and demographic model have been selected, it is crucial to investigate the temporal signal existing in a dataset. The most robust method existing so far for this purpose is the date-randomization test (DRT, see section 1 & Figure 2 of the main document and Duchêne et al. (2015b) for more details). The function “RandomDates” from the R package TipDatingBeast and the scripts attached to Murray *et al.* (2015) allow generating XML-formatted input files in which tip dates have been shuffled among all the taxa (see Table 1 of the main document). For datasets failing to pass the DRT, the sampling and/or the sequencing strategy should be modified to widen the sampling time window (e.g. by including older samples, if possible) and/or increasing the number of sites in the alignment (e.g. by sequencing more nucleotides, if possible). Investigating the temporal signal of a data set is crucial because even in the absence of any signal in the data, a result driven by the prior will be obtained, which can lead to misinterpretations. An alternative way to check for the presence of sufficient temporal signal in the data is to sample the MCMC from the prior only (i.e. ignoring the data) and to compare the results obtained with the standard analyses (see this link for how to perform this analysis: <http://beast.bio.ed.ac.uk/Sampling-the-Prior>). Even if a dataset passes the DRT, it is still possible that the dataset used to tip-calibrate the tree includes one or several problematic sequences. This could be the consequence of various errors such as sequencing mistakes or erroneous sampling time/radiocarbon dating. The “leave-one-out-cross-validation” test aims to detect samples that might induce such systematic bias and should thus be performed. The R package TipDatingBeast (Table 1 of the main document) contains functions to assist with the implementation of this test. Finally, as recently suggested by Emerson et al. (2015), it is relevant to test for the agreement between the topologies of tip-date-constrained and unconstrained trees as misestimated topologies may result in upwardly biased estimates of rates.

**E) Performing the tip-dating analysis, interpreting and extracting the results.**

When a dataset passes the DRT, which means that measurable evolutionary change has been observed, final tip-dating analyses must be run with the best partitioning schemes, substitution models, molecular clock and demographic models. The MCMC analyses should be run until convergence, that is, until they consistently sample from the most realistic parameter values. It is important to remove the first 10-20% of iterations (the burn-in), as these are generally unrealistic. It is also recommended to run independent MCMC with different starting values to check for convergence (those can be combined *a posteriori* using the LogCombiner Java tool). In BEAST, the MCMC generally generates two output files.

The first output is the Log file which contains a row for each MCMC sampling and a column for each parameter. When considered as frequency distributions, this file provides an estimate of the marginal posterior probability distribution for each parameter. This file can be inspected with the package TRACER (see the online tutorial <http://beast.bio.ed.ac.uk/Analysing-BEAST-output>). Parameter convergence is measured by the Effective Sample Size (ESS). The ESS of a parameter sampled from an MCMC is the number of effectively independent draws from the posterior distribution that the Markov chain is equivalent to. The ESS is of particular interest as it directly influences the standard deviation of the estimated mean of a parameter (the smaller the ESS the larger the standard deviation). Different ways of increasing the ESS of a parameter exist (see the online tutorial <http://beast.bio.ed.ac.uk/Increasing-ESSs>). An alternative trick not given in the latter protocol and that can be efficient in case of large datasets and/or large number of parameters is to independently estimate the tree topology and to fix it during the MCMC (See how to do this in part 2c of this document). The package TRACER allows computing parameters statistics such as the mean, median or 95% HPD. The 95% HPD stands for *highest posterior density interval* and represents the most compact interval on the selected parameter that contains 95% of the posterior probability. It can be thought of as a Bayesian analog to a confidence interval and is crucial to take into consideration when interpreting and reporting results.

The second output generated by BEAST is a Tree file which contains a row for each tree estimated during each MCMC sampling in either NEWICK or NEXUS format. This file generally contains a large number of rooted trees representing the distribution over all possible topologies. It can be used to investigate the posterior probability of specific quantities of interest when testing phylogenetic hypotheses, such as the monophyly of a particular group of organisms. The program DensiTree (Table 1 of the main document) allows visualizing such a large number of trees as a single plot. The program TreeAnnotator allows to summarize a set of trees into a consensus phylogeny and the program FigTree to visualize it (see the online tutorial <http://beast.bio.ed.ac.uk/Summarizing-BEAST-trees>). Each branch and node of such a consensus tree is generally annotated with statistics (mean, median, 95% HPD) for various parameters such as rate of evolution, branch length, age and node posterior support. The R package PHYLOCH (Table 1 of the main docment) allows extracting branch and node specific information from a consensus tree in order to perform statistical tests (one may for instance want to test if the rate of evolution in a clade is different from the one in another clade).

**2: Manual modifications to perform to the xml-input files**

The following tips are given for the version 1.x of the BEAST software.

**2a: Import SNP alignment only**

It is generally not advisable to only use variable sites when performing phylogenetic inferences. However, in BEAST, there is an easy way to do that by specifying the number of constant sites (these could even be relatively approximate, i.e. the overall base frequency times the sequence length minus the number of variable sites). The user needs to load the variable (SNP) sites as normal in BEAUti, generate the BEAST XML and then edit it as follows:

Look for the pattern list:

*<patterns id="patterns" from="1" every="1" >
               <alignment idref="alignment"/>
       </patterns>*

and replace it with this:

*<mergePatterns id="patterns">
               <patterns from="1" every="1">
                       <alignment idref="alignment"/>
               </patterns>

               <constantPatterns>
                       <alignment idref="alignment">
                       <counts>
                               <parameter value="555 434 543 432"/>
                       </counts>
               </constantPatterns>
       </mergePatterns>*

Where « *555 434 543 432 »* are the counts of invariant A, C, G & T sites, respectively.

**2b: Apply different coalescent tree priors to different clades in a single analysis**

In most inferences, one tree prior is generally sufficient since a single biological/demographic process responsible for generating the pattern of lineage divergences can be assumed. In some cases however, it can be hypothesized that several individuals or species have experienced significant different demographic histories. In BEAST, it is possible to model this process by applying separate tree prior models to different clades of the tree. To do so the user need to edit the xml as follows:

Step 1) In the taxa bloc, create new taxa to group the individuals with the same tree prior together. In this example let’s assume we want to apply three different tree priors.

*</taxa>*

*<taxa id="Subspecies1">*

*<taxon idref="Individual1"/>*

*<taxon idref="Individual2"/>*

*<taxon idref="Individual3"/>*

*<taxon idref="Individual4"/>*

*<taxa id="Subspecies2">*

*<taxon idref="Individual5"/>*

*<taxon idref="Individual6"/>*

*<taxon idref="Individual7"/>*

*<taxa id="Subspecies3">*

*<taxon idref="Individual8"/>*

*<taxon idref="Individual9"/>*

*<taxon idref="Individual10"/>*

*</taxa>*

Step 2) In the tree prior bloc, add as many new tree prior models as required. Then in the “generate a coalescent likelihood bloc”, link those models to the taxa defined earlier(we assume here we want the clade 1 to follow an exponential growth model and clades 2 and 3 to follow two different constant size models, with independent parameter values).

*<exponentialGrowth id="exponential" units="years">*

*<populationSize>*

*<parameter id="exponential.popSize" value="1.0" lower="0.0"/>*

*</populationSize>*

*<growthRate>*

*<parameter id="exponential.growthRate" value="0.0"/>*

*</growthRate>*

*</exponentialGrowth>*

*<constantSize id="constant_1" units="years">*

*<populationSize>*

*<parameter id="constant.popSize.1" value="1.0" lower="0.0"/>*

*</populationSize>*

*</constantSize>*

*<constantSize id="constant_2" units="years">*

*<populationSize>*

*<parameter id="constant.popSize.2" value="1.0" lower="0.0"/>*

*</populationSize>*

*</constantSize>*

*<!-- Generate a coalescent likelihood -->*

*<coalescentLikelihood id="coalescent1">*

*<model>*

*<exponentialGrowth idref="exponential"/>*

*</model>*

*<populationTree>*

*<treeModel idref="treeModel"/>*

*</populationTree>*

*<include>*

*<taxa idref=" Subspecies1"/>*

*</include >*

*</coalescentLikelihood>*

*<coalescentLikelihood id="coalescent2">*

*<model>*

*<constantSize idref="constant.1"/>*

*</model>*

*<populationTree>*

*<treeModel idref="treeModel"/>*

*</populationTree>*

*<include>*

*<taxa idref=" Subspecies2"/>*

*</include >*

*</coalescentLikelihood>*

*<coalescentLikelihood id="coalescent3">*

*<model>*

*<constantSize idref="constant.2"/>*

*</model>*

*<populationTree>*

*<treeModel idref="treeModel"/>*

*</populationTree>*

*<include>*

*<taxa idref=" Subspecies3"/>*

*</include >*

*</coalescentLikelihood>*

Step 3) In the operator bloc, add the new parameter to make sure there are included in the MCMC optimization.

*<!-- Define operators -->*

*<operators id="operators">*

*<scaleOperator scaleFactor="0.75" weight="3">*

*<parameter idref="exponential.popSize"/>*

*</scaleOperator>*

*<randomWalkOperator windowSize="1.0" weight="3">*

*<parameter idref="exponential.growthRate"/>*

*</randomWalkOperator>*

*<scaleOperator scaleFactor="0.75" weight="3">*

*<parameter idref="constant.popSize.1"/>*

*</scaleOperator>*

*<scaleOperator scaleFactor="0.75" weight="3">*

*<parameter idref="constant.popSize.2"/>*

*</scaleOperator>*

*</operators>*

Step 4) Make sure to also include the new parameters in the “Define MCMC” bloc to fix priors, and in the “log to file” bloc to sample them during the MCMC.

*<!-- Define MCMC -->*

*<prior id="prior">*

*<uniformPrior lower="0.001" upper="100.0">*

*<parameter idref="exponential.popSize"/>*

*</uniformPrior>*

*<laplacePrior mean="0.0" scale="12.0">*

*<parameter idref="exponential.growthRate"/>*

*</laplacePrior>*

*<uniformPrior lower="0.001" upper="50.0">*

*<parameter idref="constant.popSize.1"/>*

*</uniformPrior>*

*<uniformPrior lower="0.001" upper="50.0">*

*<parameter idref="constant.popSize.2"/>*

*</uniformPrior>*

*<coalescentLikelihood idref="coalescent1"/>*

*<coalescentLikelihood idref="coalescent2"/>*

*<coalescentLikelihood idref="coalescent3"/>*

*</prior>*

*<log id="fileLog" logEvery="2500" fileName="Scheme1.log.txt" overwrite="false">*

*<parameter idref="exponential.popSize"/>*

*<parameter idref="exponential.growthRate"/>*

*<parameter idref="constant.popSize.2"/>*

*<parameter idref="constant.popSize.1"/>*

*<coalescentLikelihood idref="coalescent1"/>*

*<coalescentLikelihood idref="coalescent2"/>*

*<coalescentLikelihood idref="coalescent3"/>*

*</log>*

**2c: Fix the tree topology during BEAST inference**

Even though tree topology and other genetic/demographic parameters should in theory be conjointly estimated, there are situations where the user may want to fix the tree topology during phylogenetic inferences (this may for instance help to reach convergence in the case of analyses including a very high number of parameters). To do so in BEAST, the user needs to edit the xml as follows:

Step 1) Fix the initial topology

Delete the statement controlling the initial tree construction and insert the following bloc to fix the initial topology to the one given in newick format

*<newick id="startingTree">*

*((((((((Tarsius_syrichta,Lemur_catta),Saimiri_sciureus),(M_sylvanus,(M_fascicularis,(M_mulatta,Macaca_fuscata)))),Hylobates),Pongo),Gorilla),Pan),Homo_sapiens);*

*</newick>*

Step 2) Keep this initial topology constant during MCMC

To keep the initial topology constant, inactivate (with <!—and -->) all the operators acting on the treeModel.

Those are <narrowExchange>, <wideExchange>, <wilsonBalding> and <subtreeSlide>.

*<operators id="operators">*

*<!--*

*<subtreeSlide size="91.0" gaussian="true" weight="15">*

*<treeModel idref="treeModel"/>*

*</subtreeSlide>*

*<narrowExchange weight="15">*

*<treeModel idref="treeModel"/>*

*</narrowExchange>*

*<wideExchange weight="3">*

*<treeModel idref="treeModel"/>*

*</wideExchange>*

*<wilsonBalding weight="3">*

*<treeModel idref="treeModel"/>*

*</wilsonBalding>*

*-->*

*</operators>*

**References cited**

Axelsson E, Willerslev E, Gilbert MTP, Nielsen R (2008) The effect of ancient DNA damage on inferences of demographic histories. *Molecular Biology and Evolution* **25**, 2181-2187.

Baele G, Lemey P, Bedford T*, et al.* (2012) Improving the Accuracy of Demographic and Molecular Clock Model Comparison While Accommodating Phylogenetic Uncertainty. *Molecular Biology and Evolution* **29**, 2157-2167.

Der Sarkissian C, Allentoft ME, Avila-Arcos MC*, et al.* (2015) Ancient genomics. *Philosophical transactions of the Royal Society of London. Series B, Biological sciences* **370**, 20130387-20130387.

Didelot X, Wilson DJ (2015) ClonalFrameML: Efficient Inference of Recombination in Whole Bacterial Genomes. *Plos Computational Biology* **11**.

Drummond A, Bouckaert R (2015) *Bayesian evolutionary analysis with BEAST 2* Cambridge University Press.

Duchêne D, Duchêne S, Holmes EC, Ho SYW (2015) Evaluating the adequacy of molecular clock models using posterior predictive simulations. *Molecular Biology and Evolution*.

Emerson BC, Alvarado-Serrano DF, Hickerson MJ (2015) Model misspecification confounds the estimation of rates and exaggerates their time dependency. *Molecular Ecology* **24**, 6013-6020.

Green RE, Malaspinas A-S, Krause J*, et al.* (2008) A complete neandertal mitochondrial genome sequence determined by high-throughput Sequencing. *Cell* **134**, 416-426.

Heath TA, Huelsenbeck JP, Stadler T (2014) The fossilized birth-death process for coherent calibration of divergence-time estimates. *Proceedings of the National Academy of Sciences of the United States of America* **111**, E2957-E2966.

Heled J, Drummond AJ (2012) Calibrated Tree Priors for Relaxed Phylogenetics and Divergence Time Estimation. *Systematic Biology* **61**, 138-149.

Ho SYW, Larson G, Edwards CJ*, et al.* (2008) Correlating Bayesian date estimates with climatic events and domestication using a bovine case study. *Biology Letters* **4**, 370-374.

Ho SYW, Phillips MJ (2009) Accounting for Calibration Uncertainty in Phylogenetic Estimation of Evolutionary Divergence Times. *Systematic Biology* **58**, 367-380.

Huson DH, Bryant D (2006) Application of phylogenetic networks in evolutionary studies. *Molecular Biology and Evolution* **23**, 254-267.

Jonsson H, Ginolhac A, Schubert M, Johnson PLF, Orlando L (2013) mapDamage2.0: fast approximate Bayesian estimates of ancient DNA damage parameters. *Bioinformatics* **29**, 1682-1684.

Lanfear R, Calcott B, Ho SYW, Guindon S (2012) PartitionFinder: Combined Selection of Partitioning Schemes and Substitution Models for Phylogenetic Analyses. *Molecular Biology and Evolution* **29**, 1695-1701.

Martin DP, Murrell B, Golden M, Khoosal A, Muhire B (2015) RDP4: Detection and analysis of recombination patterns in virus genomes. *Virus Evolution*.

McVean G, Awadalla P, Fearnhead P (2002) A coalescent-based method for detecting and estimating recombination from gene sequences. *Genetics* **160**, 1231-1241.

Murray GGR, Wang F, Harrison EM*, et al.* (2015) The effect of genetic structure on molecular dating and tests for temporal signal. *Methods in Ecology and Evolution*.

Paabo S (1989) Ancient DNA: extraction, characterization, molecular cloning, and enzymatic amplification. *Proceedings of the National Academy of Sciences of the United States of America* **86**, 1939-1943.

Rambaut A, Ho SYW, Drummond AJ, Shapiro B (2009) Accommodating the Effect of Ancient DNA Damage on Inferences of Demographic Histories. *Molecular Biology and Evolution* **26**, 245-248.

Rieux A, Eriksson A, Li M*, et al.* (2014) Improved Calibration of the Human Mitochondrial Clock Using Ancient Genomes. *Molecular Biology and Evolution* **31**, 2780-2792.

Sawyer S, Krause J, Guschanski K, Savolainen V, Paeaebo S (2012) Temporal Patterns of Nucleotide Misincorporations and DNA Fragmentation in Ancient DNA. *Plos One* **7**.

Shapiro B, Ho SYW, Drummond AJ*, et al.* (2011) A Bayesian Phylogenetic Method to Estimate Unknown Sequence Ages. *Molecular Biology and Evolution* **28**, 879-887.

Stadler T (2009) On incomplete sampling under birth-death models and connections to the sampling-based coalescent. *Journal of Theoretical Biology* **261**, 58-66.

Stadler T (2010) Sampling-through-time in birth-death trees. *Journal of Theoretical Biology* **267**, 396-404.

Wiens JJ, Moen DS (2008) Missing data and the accuracy of Bayesian phylogenetics. *Journal of Systematics and Evolution* **46**, 307-314.
